# Altered White Matter Integrity after Mild to Moderate Traumatic Brain Injury

**DOI:** 10.3390/jcm8091318

**Published:** 2019-08-27

**Authors:** Eunkyung Kim, Han Gil Seo, Hyun Haeng Lee, Seung Hak Lee, Seung Hong Choi, Roh-Eul Yoo, Won-Sang Cho, Amy K. Wagner, Byung-Mo Oh

**Affiliations:** 1 Department of Rehabilitation Medicine, Seoul National University Hospital, Seoul 03080, Korea; 2 Department of Rehabilitation Medicine, Konkuk University School of Medicine and Konkuk University Medical Center, Seoul 03080, Korea; 3 Department of Rehabilitation Medicine, Asan Medical Center, University of Ulsan College of Medicine, Seoul 03080, Korea; 4 Department of Radiology, Seoul National University College of Medicine, Seoul 03080, Korea; 5 Department of Radiology, Seoul National University Hospital, Seoul 03080, Korea; 6 Department of Neurosurgery, Seoul National University Hospital, Seoul 03080, Korea; 7 Department of Physical Medicine and Rehabilitation, University of Pittsburgh, Pittsburgh 15260, PA, USA

**Keywords:** traumatic brain injury, diffusion tensor imaging, postural balance, memory deficit

## Abstract

(1) Background: White matter changes among individuals with mild-to-moderate traumatic brain injury (TBI) may be sensitive imaging markers reflecting functional impairment, particularly in the context of post-concussion syndrome. The objective of this study was to examine the altered white matter integrity in mild-to-moderate TBI patients compared with age-matched normal controls. (2) Methods: Diffusion tensor imaging data from 15 individuals with TBI and 15 control subjects were retrospectively obtained. We investigated and compared white matter integrity in both groups, with regard to fractional anisotropy (FA), radial diffusivity (RD), and axial diffusivity (AD) and examined the relationship with cognitive dysfunction and impaired balance in patients. (3) Results: In comparison with controls, the TBI patients had significantly decreased FA as well as increased RD, in the right corticospinal tract. Decreased RD was observed in the left cerebellar area near the middle cerebellar peduncle. Decreased AD was observed in the left inferior cerebellar peduncle, showing positive correlation with poor balance control. We observed decreased FA and increased AD in the left superior longitudinal fasciculus showing positive and negative correlation, respectively, with cognitive function in the TBI group. (4) Conclusions: Altered white matter integrity in mild-to-moderate TBI cases may be indicative of cognitive dysfunction and impaired balance.

## 1. Introduction

Traumatic brain injury (TBI), notwithstanding a recent increase in incidence, remains a largely underestimated brain injury, despite occurring in more than 1.5 million individuals in the United States [1], and mild-to-moderate TBI occurs in 90% of all cases [2]. Symptoms may vary and include reduced concentration, memory deficits, and impaired balance [3,4]. The majority of patients recover from these symptoms within 3 months [5] but, for some individuals, symptoms following TBI can persist for more than a year, referred to as post-concussion syndrome (PCS) [6]. There are no well-validated diagnostic tools to predict the likelihood of each patient experiencing PCS [7]. Given this, understanding the concomitant brain changes, associated with TBI-induced dysfunctions and disabilities is an important step toward facilitating rehabilitation responsivity in these patients.

To detect brain changes after TBI, diverse imaging techniques have been utilized [8,9] including computed tomography (CT), due to its rapid availability and cost-effectiveness. Nevertheless, most individuals with mild-to-moderate TBI exhibit no abnormal findings on CT scans. On the other hand, diffusion tensor imaging (DTI) is much more effective in detecting axonal changes in the brain [9], a characteristic observed after mild-to-moderate TBI [10].

DTI with magnetic resonance imaging (MRI) measures the diffusion of water molecules within a tissue and estimates their direction; which is particularly useful in evaluating the integrity of the white matter, where water is abundant [11]. Using DTI, previous studies have suggested that altered diffusion-based anisotropy measures, including fractional anisotropy (FA), axial diffusivity (AD), and radial diffusivity (RD) are observed among individuals with mild-to-moderate TBI and these findings have been associated with cognitive dysfunction and physical disabilities commonly observed after TBI [12,13]. On the other hand, another study reported that the diffusion parameters observed during the acute phase following mild TBI were not abnormal compared to age- and sex-matched controls [14].

The aim of our study was to investigate white matter changes among individuals with mild-to-moderate TBI. Based on previous studies, we hypothesized that (1) there are specific white matter regions particularly vulnerable to TBI, and (2) white matter changes in these regions would be significantly associated with clinical measures of cognitive or physical dysfunction. For example, previous studies have reported decreased FA in the frontal lobe [15], temporal lobe [16], and cerebellum [17] of TBI patients, which were significantly correlated with deficits in executive functions, memory, and balance, respectively. By applying tract-based spatial statistics (TBSS), a popular voxel-wise analysis of the whole brain [18], we analyzed the whole brain white matter to overcome the limitations of previous regions-of-interest (ROIs) studies [12,19]. We estimated FA, AD, and RD, and compared these values between the TBI and control groups. We also determined the correlation coefficients between the diffusion-based anisotropy measures and the sub-scores in cognitive testing as well as scores in balance testing.

## 2. Materials and Methods

### 2.1. Participants

Patients with mild-to-moderate TBI from the database of the Department of Rehabilitation at Seoul National University Hospital were enrolled in the study. They visited the hospital from October 2016 to July 2017. The criteria for diagnosis included the Glasgow Coma Scale (GCS) score, the duration of loss of consciousness (LOC), and the duration of post-traumatic amnesia (PTA). Individuals with GCS scores of 13–15, duration of LOC shorter than 30 min, and duration of PTA shorter than 24 h were identified as having mild TBI. Individuals with GCS scores of 9–12, duration of LOC shorter than 24 h, and duration of PTA shorter than one week were identified as having moderate TBI [20]. Fifteen patients in the database met the criteria for mild-or-moderate TBI (4 men/11 women, mean age: 49.1 ± 10.5 years). The brain MRI data were obtained during the post-acute stage, i.e., at least 4 weeks after injury (range: from 29 to 1380 days, 413.6 ± 550.8 days). 

Patients with mild headache or migraine, without history of neurological disease or head trauma, were included in the control group (4 men/11 women, mean age: 49.1 ± 10.5 years). They showed negative or nonspecific mild T2 high signal intensity findings in MRI.

This was a retrospective study, approved by the Institutional Review Board of the Seoul National University Hospital (1708-153-879). We adopted a retrospective study design because we have treated patients with mild to moderate traumatic brain injury from 2016 in an outpatient clinic using a consistent clinical protocol. In this protocol, the symptoms and status of the patients were routinely assessed. We confirmed that all methods were performed in accordance with the relevant guidelines and regulations. Written informed consent was not obtained from the participants because of the retrospective design of this study. The patients’ brain imaging data were collected as part of the standard clinical evaluation in 2016 and 2017.

### 2.2. Evaluation of Post-concussion Symptoms, Cognitive and Physical Function

Several clinical questionnaires were used to assess the patients: The Rivermead Post-Concussion Symptom Questionnaire (RPCSQ), the Extended Glasgow Outcome Scale (GOSE), the Galveston Orientation and Amnesia Test (GOAT), and the Computerized NeuroCognitive Function Test (CNT 40^®^; MaxMedica, Seoul, Korea). The conducted CNT comprised four different tests, including an auditory continuous performance test, a verbal learning test, a digit span test, and a card-sorting test. The auditory continuous performance test assessed continuous concentration on auditory stimulation by counting correct responses and commission errors. The verbal learning test assessed verbal memory performance after the subject heard 15 words. Outcome measures included the following: The number of words recalled in the first trial, the number of words recalled in the fifth trial, and the delayed recall, i.e., the number of words recalled after 20 min. The digit span test assessed memory function both forward and backward. The card-sorting test assessed executive function. Balance was assessed using a posturography device (Tetrax®, Sunlight Medical Ltd., Tel Aviv, Israel), which measures the center of pressure (COP) under different conditions [21]. During each condition, the patient stands on the Tetrax instrument for 32 s, while attempting to maintain body balance. The outcome measures from this test were length, and length-over-area of the COP sway, while an individual stood on a foam rubber pillow with either open or closed eyes. High scores were indicative of poor balance control [21]. Detailed characteristics of the patients are listed in Table 1 and Table 2.

### 2.3. Image Acquisition

Brain imaging data were obtained using a Discovery MR750w 3.0T scanner (General Electric Healthcare, Milwaukee, WI, USA). Structural T1 images were acquired using a sagittal three-dimensional fast spoiled gradient recalled echo acquisition protocol with the following parameters: Image matrix = 256 mm × 230 mm; voxel size = 1 mm^3^; repetition time (TR) = 8.5 ms for 11 patients and 11 controls, and 8.6 ms for 4 patients and 4 controls; echo time (TE) = 3.2 ms; field of view (FOV) = 256 mm. The number of sagittal slices spanning the whole brain differed slightly between subjects: 162, 166, 170, 174, 178, or 182 slices were acquired depending on the brain size. DTI were acquired using a single shot pulsed gradient dual echo acquisition protocol with the following parameters: axial acquisition = 128 mm × 128 mm; voxel size = 0.9 × 0.9 × 4 mm^3^; TR = 8000 ms. TE differed slightly between subjects, ranging from 76.1 to 85.0 ms. The FOV was 240 mm, and the flip angle was 90°. The number of axial slices of the whole brain differed slightly between subjects: 34, 35, 36, or 37 slices were acquired, depending on the brain size. All DTI images had 15 distributed orientations at a b-value of 1000 s/mm^2^, with one b = 0 image.

### 2.4. Data Preprocessing

All DTI images were preprocessed using the FSL software (version 5.0.10, http://www.fmrib.ox.ac.uk/fsl) [22]. After a visual inspection of the DTI images, eddy current correction was performed to control both eddy current-induced distortion and subject movement detected within the brain image [23]. The corrected data were skull stripped. Estimation of the diffusion tensor matrix at each voxel was performed by FMRIB’s Diffusion Toolbox (FDT). The diffusion parameters, FA, AD, and RD were estimated from the matrix using the three eigenvalues λ_1_, λ_2_, and λ_3_. FA is the normalized variance of the three eigenvalues; thus, it is used to represent relative anisotropy compared with AD and RD, which reflect more specific characteristics. AD is the first eigenvalue representing diffusivity parallel to the axon. RD is the average of the second and third eigenvalues, representing diffusivity perpendicular to the axon.

### 2.5. Statistical Analyses

We first compared the mean age of the two groups using a Wilcoxon’s rank-sum test. The relationship between age and cognitive and behavioral parameters was investigated using *S*pearman’s correlation analysis, including the RPCSQ scores, the CNT sub-scores, and the balance scores. The relationship of the cognitive and behavioral parameters with the duration after the injury until the brain imaging were also investigated using *S*pearman’s correlation analysis.

#### Comparing Diffusion-Anisotropy Findings between Groups and Estimating Correlation with the Cognitive and Physical Parameters

TBSS is one of the voxel-wise statistical approaches, which analyses anisotropic diffusion of water in white matter tracts by projecting onto the alignment-invariant tract representation [18]. Nonlinear registration of all FA, AD, and RD images into a standard space followed by projection onto the mean FA skeleton image increases the sensitivity of the analysis [18]. We compared the FA, AD, and RD images of the patients with those of the controls using the FSL’s Randomise Tool with age as a nuisance variable based on 10,000 non-parametric permutations. Statistical significance was set at *p* < 0.001 with a cluster of an extent threshold of *k* > 10 voxels. Significant differences between the two groups were observed mostly using this approach rather than the threshold-free cluster enhancement, which provide cluster-based thresholding [24].

Following this process, we extracted FA, AD, and RD values from the voxels showing significant differences between the TBI and control groups, and we then calculated the *S*pearman’s correlation coefficient between the FA, AD, and RD and the cognitive or physical parameters. The cognitive parameters analyzed in this study were the CNT sub-scores, including scores in the auditory continuous performance, verbal learning, digit span, and card sorting. The physical parameters analyzed in this study were the balance scores, including length, and length-over-area of COP sway under two alternate conditions (i.e., standing on a foam-rubber pillow with open or closed eyes). Statistical significance was detected using the *S*pearman’s correlation coefficient.

## 3. Results

There was no significant difference in age between the two groups (*p* > 0.05). No significant correlation was detected between age and RPCSQ, CNT sub-scores, or balance testing performance in the TBI group (*p* > 0.05), except for negative correlation with the performance in the card-sorting test (*r* = −0.539 and *p* = 0.047). There was no significant correlation between duration between the time of injury and brain scanning and RPCSQ, CNT sub-scores, or balance testing performance in the TBI group (*p* > 0.05), except for negative correlation with the auditory continuous performance test, counting commission error (*r* = −0.552 and *p* = 0.041) (Table 3). 

### 3.1. Altered Diffusion-Based Anisotropy Findings Observed in the Mild-to-Moderate TBI Group 

**Figure 1:**
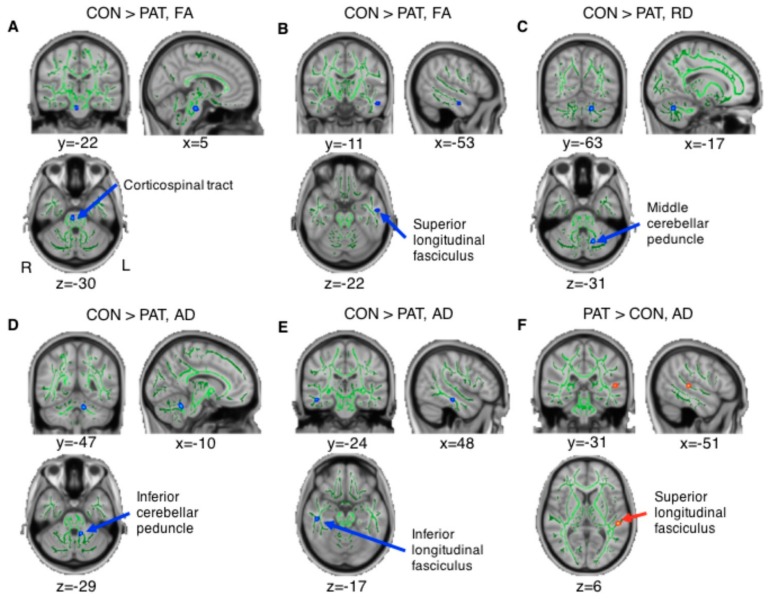
Significantly different diffusion-based anisotropy findings observed in TBI patients compared with controls (uncorrected *p* < 0.001 with a cluster of an extent threshold of *k* > 10 voxels). The figure was visualized on a standard MNI152_T1 brain template with white matter skeleton (green). The locations visualized as red and blue indicate the following regions: (**A**) right corticospinal tract, (**B**) left superior longitudinal fasciculus, (**C**) left middle cerebellar peduncle, (**D**) left inferior cerebellar peduncle, (**E**) right inferior longitudinal fasciculus, and (**F**) left superior longitudinal fasciculus. TBI patients showing (a,b) decreased FA, (c) decreased RD, (d,e) decreased AD, and (f) increased AD in the brain areas. For visualization, group differences found in the TBSS were “thickened” and “filled out” into the local tracts of mean FA track skeleton by using the tbss_fill script. Abbreviations: CON, controls; PAT, patients; TBI, traumatic brain injury, FA, fractional anisotropy; RD, radial diffusivity; AD, axial diffusivity; TBSS, tract-based spatial statistics.

The TBI group showed significantly decreased FA as compared with controls in the right corticospinal tract and left superior longitudinal fasciculus (Figure 1A,B), and exhibited significantly decreased RD in the left cerebellar area close to the middle cerebellar peduncle (Figure 1C). Significantly decreased AD in patients was observed in the left inferior cerebellar peduncle, and right inferior longitudinal fasciculus (Figure 1D,E). Significantly increased AD was observed in TBI patients in the left superior longitudinal fasciculus (Figure 1F). The voxel coordinates of local maxima are listed in Table 4. Based on the Johns Hopkins University white matter (JHU-WM) Tractography Atlas and the Johns Hopkins University-The International Consortium for Brain Mapping (JHU-ICBM)-DTI-81 WM Labels provided by the FSL, we defined the region of the voxels showing maximal significance.

We also found similar changes in the diffusion-based anisotropy findings in the contralateral areas using a more liberal threshold (*p* < 0.005), specifically in the cerebellar areas, which implies that similar brain changes occurred bilaterally. For example, the bilateral inferior cerebellar peduncles showed decreased FA in the TBI group. The bilateral middle cerebellar peduncle showed decreased RD whereas the bilateral inferior cerebellar peduncles showed decreased AD in TBI patients (Figure S1).

### 3.2. Correlations Between the Altered Diffusion-based Anisotropy Findings and Scores in CNT and Balance Testing

**Figure 2:**
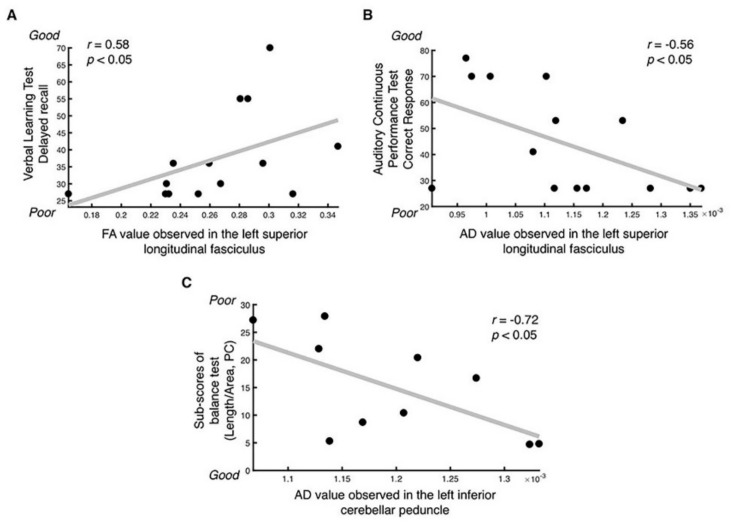
Significant correlation between FA or AD and the score of cognitive test (i.e., CNT) or balance test. (**A**) Significant positive correlation between FA of the left superior longitudinal fasciculus and the sub-score of CNT, i.e., the number of words recalled after a 20-min delayed period was observed (*S*pearman’s correlation coefficient *r* = 0.58 and *p* < 0.05). (**B**) Significant negative correlation between AD of the left superior longitudinal fasciculus and the sub-score in CNT, correct response of the auditory continuous performance test, was also observed (*S*pearman’s correlation coefficient *r* = −0.56 and *p* < 0.05). (**C**) Significant positive correlation between AD of the left inferior cerebellar peduncle and score of the balance test, i.e., the length-over-area of movement of the COP in the PC, was observed (*S*pearman’s correlation coefficient *r* = −0.72 and *p* < 0.05). Abbreviations: FA, fractional anisotropy; AD, axial diffusivity; CNT, Computerized NeuroCognitive Function Test; COP, center of pressure; PC, closed eyes.

Given the results showing significant group differences in diffusion-based anisotropy, we calculated the correlation coefficient between the diffusion-based anisotropy findings, CNT sub-scores, and the balance testing scores. Among the 6 identified regions, the FA observed in the left superior longitudinal fasciculus, which was decreased in the TBI group (Figure 1B), showed a significantly positive correlation with the cognitive testing sub-score number of words recalled after a 20-min delayed period (*S*pearman’s *r* = 0.58, *p* = 0.03, Figure 2A). Moreover, the AD observed in the left superior longitudinal fasciculus, which was increased in the TBI group (Figure 1F), showed a significant negative correlation with the cognitive testing sub-score correct response in the auditory continuous performance test (*S*pearman’s *r* = -0.56, *p* = 0.04, Figure 2B). In addition, the AD observed in the left inferior cerebellar peduncle, which was decreased in the TBI group compared with the control group (Figure 1D), showed a significant negative correlation with the balance testing results, specifically the length-over-area of the COP in the eyes-closed condition (*S*pearman’s *r* = –0.72, *p* = 0.02, and Figure 2C) indicating that the decreased AD was associated with poor balance performance. 

## 4. Discussions

In the current study, we investigated the white matter changes detected in a mild-to-moderate TBI group and found several differences, as compared with controls. We detected decreased FA and RD, and both decreased and increased AD in several white matter tracts in the TBI group (*p* < 0.001). More specifically, decreased AD was associated with impaired balance. The decreased FA and increased AD observed in the TBI group were associated with poor memory and attentional performance. With a less stringent significant threshold, similar results were also observed in the contralateral side of the brain (*p* < 0.005) after comparison between the two groups. These findings indicate that our results may be related to a higher vulnerability of these white matter tracts to TBI, irrespective of the lesion side. Age was not a factor affecting the results since the average age of the two groups was not significantly different, and the age of the TBI patients was not significantly correlated with the behavioral parameters in this study (*p* > 0.05), except for the digit span test in which we did not find any significant association with the altered diffusion-based anisotropy findings. Duration between the time of injury and brain scanning was also not a factor affecting the results of this study, since it was not significantly correlated with the behavioral parameters (*p* > 0.05), except for the auditory continuous performance test, counting commission error, in which we did not find any significant association with the altered diffusion-based anisotropy findings. In addition, duration was not significantly correlated with the FA, AD, and RD from the white matter areas, showing significant differences in comparison of the control group. For example, the FA value from the voxel of the right corticospinal tract and the left superior longitudinal fasciculus listed on Table 4 was not correlated with the duration (*r* = 0.018 and *p* = 0.954, and *r* = 0.132 and *p* = 0.639). Below, we refer to the possible causes underlying the alterations of diffusion-based anisotropy findings in this TBI group and discuss possible factors that might account for the associations observed between changes in the brain and balance or cognitive deficits.

### 4.1. Decreased FA Observed after TBI and Its Relationship with Cognitive Function

In previous studies involving individuals with TBI, altered anisotropy was observed in most of the white matter tracts, and decreased FA was the primary finding, specifically after the acute phase of injury [9]. We found decreased FA in the right corticospinal tract at the level of the brainstem and the left superior longitudinal fasciculus in the temporal lobe in the TBI group. Decreased FA may be caused by decreased AD or increased RD. AD and RD are used separately to further distinguish the complex origins of anisotropy [25,26]. Alterations in AD are known to be caused by axonal morphological changes including axonal density or caliber [27,28]. Alterations with RD are known to be caused by myelin changes [25,29]. Here, we found rather contradictory results, i.e., increased regional changes in AD of the left superior longitudinal fasciculus at the more superior part of the brain, but no regional changes in RD (*p* < 0.001). These results indicate that the decreased FA observed in the left superior longitudinal fasciculus may be associated with axonal morphological changes rather than myelin changes. However, the interpretation of these observations is difficult because the specific location was different between the decreased FA and increased AD. On the other hand, when we used a lenient threshold (*p* < 0.005), there was increased RD in the TBI group in the exact location of the right corticospinal tract (Table S1 and Figure S2) indicating that the decreased FA observed in the right corticospinal tract is associated with demyelination.

To identify the underlying mechanism affecting white matter integrity after mild-to-moderate TBI, we estimated the correlation coefficient between the diffusion-based anisotropy findings and CNT sub-scores or balance-testing scores. Opposite correlation patterns were found between decreased FA and increased AD of the superior longitudinal fasciculus with CNT sub-scores (Figure 2A,B). In detail, the FA of the left superior longitudinal fasciculus showed a significant positive correlation with the number of words recalled after the 20-min delay period, while the AD in the left superior longitudinal fasciculus showed a significant negative correlation with the correct response in the auditory continuous performance test. These findings suggest that the diffusion anisotropy value of the left superior longitudinal fasciculus, which is similar to that of control subjects, is indicative of preserved memory and attention after mild-to-moderate TBI. The superior longitudinal fasciculus is one of the major fiber bundles connecting the frontal with the temporal, parietal, and occipital areas, responsible for a variety of functions such as language, and maintenance of attention [30]. and associated with verbal working memory performance in healthy and schizophrenic subjects [31].

Based on a previous study suggesting association between the decreased FA of the corticospinal tract and poor postural control of the body in mild TBI patients [17], we hypothesized that the decreased FA in the right corticospinal tract may be correlated with the balance test scores obtained from the patients. However, we did not find a significant correlation. Further studies will be needed to demonstrate the relationship between altered diffusion anisotropy observed in the corticospinal tract and postural balance control observed in patients. The corticospinal tract is related with motor function, specifically movement of the distal extremities [32], and degenerating myelinated axons of the corticospinal tract have also been observed in an experimental TBI model [33].

### 4.2. Decreased RD and AD Observed after TBI and Their Relationship with Postural Balance Control

We found significantly decreased RD and AD in the TBI group as compared with controls, in the cerebellum for both measures, and in the right inferior longitudinal fasciculus for the AD. The cerebellar area is near the left middle cerebellar peduncle in the case of RD, and corresponds to the left inferior cerebellar peduncle in the case of AD. The cerebellum is recognized as the center of motor learning [34], and its dysfunction may be related with the impaired balance control [35]. In previous studies, three mild TBI patients with lower capability of maintaining balance showed discontinuation of the inferior cerebellar peduncle, with decreased number of fibers [36], and the FA of the inferior cerebellar peduncle positively correlated with the improvement of balance in moderate-to-severe TBI patients [37]. Therefore, these results suggest that myelin or axonal morphological changes of the middle and inferior cerebellar peduncles may be associated with the balance ability in the mild-to-moderate TBI group. To test this hypothesis, we estimated the correlation coefficient between altered anisotropy values and balance scores (measured by standing on a foam-rubber pillow with open or closed eyes) and identified a significant negative correlation between the AD of the inferior cerebellar peduncle and the balance scores in the closed-eyes condition (Figure 2A). As increased balance scores correspond to poorer balance control, the negative correlation indicates that TBI patients with severe deprivation of balance control exhibited decreased AD at a higher extend than individuals with TBI having relatively good balance control. The association between the balance ability of the TBI group, and the altered white matter tracts has been reported previously [36,37,38,39], but this is the first study demonstrating this relationship using diffusion-based anisotropy findings, in mild-to-moderate TBI patients. 

### 4.3. Limitations

Although we detected significant differences (decreased FA, RD, decreased/increased AD) in the TBI group, diverse factors, such as chronicity (acute, subacute, or chronic), the age of the patient, and symptom severity can also affect these results, due to retrospective aspects of the study [40]. For example, we had to include supposedly moderate as well as mild TBI patients in this study, because many of them could not remember the exact duration of their loss of consciousness and PTA. Patient characteristics and etiology of the injury were also different. In addition, using data acquired with relatively few gradient encoding directions (n = 15), in order to minimize scan time, may have led to inaccurate estimation of diffusion tensor matrix, even though our findings were consistent with those in previous TBI studies. It could have been better to consider different TE of the data [41], even though there was no significant difference between the TE of the patient group and control group (*p* = 0.724, Wilcoxon rank-sum test). Additional solid studies incorporating these factors will be necessary to validate the explanations we have proposed. In a previous animal study, immunohistochemical examination revealed that extensive axonal damage occurred in the brainstem (pons and midbrain), and the severity of coma was correlated with the extent of axonal damage in the region [42]. It is consistent with the finding of decreased FA in the right corticospinal tract in our mild to moderate traumatic brain injury patients compared to the control group. Future studies investigating cellular changes of the brain due to traumatic brain injury will be also necessary along with in vivo diffusion tensor imaging study.

## 5. Conclusions

The results of this study highlight the changes that occur in white matter microstructures in mild-to-moderate TBI patients. Cognitive and physical dysfunction with diverse sequelae showed significant association with changes observed in the brain, which implies diffusion tensor parameters can have potential as imaging biomarkers of functional recovery.

## Figures and Tables

**Table 1:** Characteristics of patients I.

ID	Diagnose	Gender	Age	GCS	LOC	PTA	Duration	RPCSQ	GOSE	GOAT
P01	mild	F	46	15	< 30 min	< 30 min	56	17	NA	NA
P02	moderate	M	57	NA	~3 h	1 day–1 week	174	9	NA	NA
P03	moderate	M	20	NA	(+) but no details available	1 day–1 week	33	23	7	64
P04	moderate^1^	M	49	15	(+) but no details available	1 day	80	29	6	80
P05	moderate	F	43	NA	1 h	1 day–1 week	1336	33	5	90
P06	moderate	F	37	NA	(+) but no details available	3 day	1334	44	5	99
P07	mild	F	53	15	(+) a few min	1 h	201	37	6	NA
P08	mild	F	53	NA	(+) but no details available	(+) but no details available	873	NA	NA	93
P09	mild	F	60	15	(+) but no details available	(+) a few min	150~180^2^	39	6	100
P10	mild	F	54	15	(+) but no details available	(+) but no details available	107	56	5	88
P11	mild	F	56	NA	< 30 min	6 h	1380	NA	NA	NA
P12	moderate	F	43	15	< 5 h	1 day-1 week	29	55	4	94
P13	mild	F	49	NA	5–10 min	3–5 min	46	NA	NA	98
P14	moderate	M	61	NA	(+) but no details available	4–5 day	111	NA	NA	79
P15	mild	F	56	15	-	1 h	31	25	7	NA

Abbreviations: Age, Age (years) at the time of brain imaging; GCS, Glasgow coma scale within 24 h after injury; LOC, Loss of consciousness; PTA, Post-traumatic amnesia; Duration, Duration (days) between the time of injury and brain scanning; RPCSQ, Rivermead post-concussion symptom questionnaire; GOSE, Extended glasgow outcome scale; GOAT, Galveston orientation and amnesia test; F, Female; NA, Not available; M, Male; (+), loss of consciousness was reportedly evident; moderate^1^, P04 was diagnosed as moderate TBI because of infarct which may have been caused by vascular injury due to trauma; 150~180^2^, P09 only remembered the month and not the day of injury. For correlation analysis, duration of P09 was considered 165 days; Scores were measured within the range from 0 to 67 days before or after brain scanning.

**Table 2:** Characteristics of patients II.

ID	Cognitive Function Test								Balance Test			
	Auditory Continuous Performance Test		Verbal Learning Test			Digit Span Test		Card Sorting Test	Standing on a Foam-Rubber Pillow with Eyes Open		Standing on a Foam-Rubber Pillow with Eyes Closed	
	Correct Response	Commission Error	A1	A5	Delayed Recall	Forward	Backward		Length	Length/Area	Length	Length/Area
P01	NA								NA			
P02	70	70	45	38	27	44	34	40	NA			
P03	70	77	45	50	55	35	47	77	NA			
P04	77	77	39	30	27	46	53	52	NA			
P05	27	27	50	54	30	44	50	58	42.3	11.3	122.8	16.7
P06	27	27	45	38	36	42	47	51	41.1	16	91.9	4.7
P07	41	45	55	70	70	34	38	36	34.9	21.6	78.4	27.9
P08	27	30	45	38	30	27	30	46	37.4	14.8	23.8	8.7
P09	70	56	45	42	27	44	47	46	29.6	38.2	39.6	27.2
P10	27	27	39	38	41	27	34	47	84.6	3.6	142.5	5.3
P11	27	27	63	42	36	44	53	64	134.9	14.5	196.2	20.4
P12	53	56	45	50	36	77	53	59	171.3	3	NA	
P13	27	27	39	27	27	27	27	8	135.4	5.9	220.3	4.8
P14	27	27	30	27	27	42	43	30	50.4	18.4	92.5	22
P15	53	77	55	54	55	49	53	46	37.8	19.5	88.6	10.4

Cognitive Function Test measures are T-scores. Abbreviations: A1, the number of words recalled in the first trial; A5, the number of words recalled in the fifth trial; NA, not available. Cognitive test scores were measured within the range from 0 to 15 days before or after brain scanning, except one patient obtained scores 51 days before brain scanning. Balance test scores were measured within the range from 0 to 11 days before or after brain scanning, except one patient obtained scores 11 days after brain scanning.

**Table 3:** Correlation coefficient with the age or duration to the RPCSQ, CNT sub-scores, or balance testing performance in the individuals with mild to moderate traumatic brain injury.

	RPCSQ	Cognitive Function Test								Balance Test			
		Auditory Continuous Performance Test		Verbal Learning Test			Digit Span Test		Card Sorting Test	Standing on a Foam-Rubber Pillow with Eyes Open		Standing on a Foam-Rubber Pillow with Eyes Closed	
		Correct Response	Commiss-Ion Error	A1	A5	Delayed Recall	For-Ward	Back-Ward		Length	Length/Area	Length	Length/Area
Age	−0.072	0.012	−0.044	−0.049	−0.211	−0.338	0.079	−0.153	−0.539^*^	−0.252	0.499	−0.146	0.592
Duration	0.118	−0.477	−0.552*	0.384	0.042	−0.095	−0.219	−0.125	0.033	−0.291	0.200	−0.030	0.152

Abbreviations: Age, Age (year) at the time of brain imaging; CNT, Computerized NeuroCognitive Function test; Duration, Duration (days) between the time of injury and brain scanning; RPCSQ, Rivermead post-concussion symptom questionnaire. *p < 0.05.

**Table 4:** Results of TBSS analyses comparing the whole brain of the two groups.

Contrast	Voxel Coordinates of Local Maxima (MNI Coordinates)			Side	Voxels	White Matter Tract	
	x	y	z			JHU-WM Tractography Atlas	JHU-ICBM-DTI-81 WM Labels
CON > PAT							
Fractional anisotropy							
	5	–22	–30	R	14	Corticospinal tract	Corticospinal tract
	–53	–11	–22	L	10	Unclassified	Superior longitudinal fasciculus
Radial diffusivity							
	–17	–63	–31	L	16	Unclassified	Unclassified
Axial diffusivity							
	–10	–47	–29	L	22	Inferior cerebellar peduncle	
	48	–24	–17	R	15	Unclassified	Inferior longitudinal fasciculus
PAT > CON							
Axial diffusivity							
	–51	–31	6	L	11	Unclassified	Superior longitudinal fasciculus

Abbreviations: TBSS, Tract-based spatial statistics; MNI, Montreal Neurological Institute; JHU-WM, the Johns Hopkins University white matter; JHU-ICBM, the Johns Hopkins University-The International Consortium for Brain Mapping; DTI, diffusion tensor imaging; CON, controls; PAT, patients; R, right; L, left. Statistical significance was set at *p* < 0.001 with a cluster of an extent threshold of *k* > 10 voxels.

## Supplementary Materials

The following are available online at https://www.mdpi.com/2077-0383/8/9/1318/s1, Figure S1: The significantly different diffusion-tensor anisotropy findings observed in the traumatic brain injury patients compared to the controls, Table S1: Results of TBSS analyses comparing RD values for the whole brain of the two groups.

## References

[1] Bazarian J.J., Veazie P., Mookerjee S., Lerner E.B. (2006). Accuracy of mild traumatic brain injury case ascertainment using ICD-9 codes. Acad. Emerg. Med..

[2] Bruns J., Hauser W.A. (2003). The epidemiology of traumatic brain injury: A review. Epilepsia.

[3] Rabinowitz A.R., Levin H.S. (2014). Cognitive sequelae of traumatic brain injury. Psychiatr. Clin. N. Am..

[4] Maskell F., Chiarelli P., Isles R. (2006). Dizziness after traumatic brain injury: Overview and measurement in the clinical setting. Brain Inj..

[5] Levin H.S., Mattis S., Ruff R.M., Eisenberg H.M., Marshall L.F., Tabaddor K., High W.M., Frankowski R.F. (1987). Neurobehavioral outcome following minor head injury: A three-center study. J. Neurosurg..

[6] Bazarian J.J., Wong T., Harris M., Leahey N., Mookerjee S., Dombovy M. (1999). Epidemiology and predictors of post-concussive syndrome after minor head injury in an emergency population. Brain Inj..

[7] Silverberg N.D., Gardner A.J., Brubacher J.R., Panenka W.J., Li J.J., Iverson G.L. (2015). Systematic review of multivariable prognostic models for mild traumatic brain injury. J. Neurotrauma.

[8] Belanger H.G., Vanderploeg R.D., Curtiss G., Warden D.L. (2007). Recent neuroimaging techniques in mild traumatic brain injury. J. Neuropsychiatry Clin. Neurosci..

[9] Eierud C., Craddock R.C., Fletcher S., Aulakh M., King-Casas B., Kuehl D., LaConte S.M. (2014). Neuroimaging after mild traumatic brain injury: Review and meta-analysis. Neuroimage Clin..

[10] Johnson V.E., Stewart W., Smith D.H. (2013). Axonal pathology in traumatic brain injury. Exp. Neurol..

[11] Alexander A.L., Lee J.E., Lazar M., Field A.S. (2007). Diffusion tensor imaging of the brain. Neurotherapeutics.

[12] Niogi S.N., Mukherjee P. (2010). Diffusion tensor imaging of mild traumatic brain injury. J. Head Trauma Rehabil..

[13] Bazarian J.J., Zhong J., Blyth B., Zhu T., Kavcic V., Peterson D. (2007). Diffusion tensor imaging detects clinically important axonal damage after mild traumatic brain injury: A pilot study. J. Neurotrauma.

[14] Ilvesmaki T., Luoto T.M., Hakulinen U., Brander A., Ryymin P., Eskola H., Iverson G.L., Ohman J. (2014). Acute mild traumatic brain injury is not associated with white matter change on diffusion tensor imaging. Brain.

[15] Lipton M.L., Gulko E., Zimmerman M.E., Friedman B.W., Kim M., Gellella E., Gold T., Shifteh K., Ardekani B.A., Branch C.A. (2009). Diffusion-tensor imaging implicates prefrontal axonal injury in executive function impairment following very mild traumatic brain injury. Radiology.

[16] Bigler E.D., McCauley S.R., Wu T.C., Yallampalli R., Shah S., MacLeod M., Chu Z., Hunter J.V., Clifton G.L., Levin H.S. (2010). The temporal stem in traumatic brain injury: Preliminary findings. Brain Imaging Behav..

[17] Caeyenberghs K., Leemans A., Geurts M., Taymans T., Linden C.V., Smits-Engelsman B.C., Sunaert S., Swinnen S.P. (2010). Brain-behavior relationships in young traumatic brain injury patients: DTI metrics are highly correlated with postural control. Hum. Brain Mapp..

[18] Smith S.M., Jenkinson M., Johansen-Berg H., Rueckert D., Nichols T.E., Mackay C.E., Watkins K.E., Ciccarelli O., Cader M.Z., Matthews P.M. (2006). Tract-based spatial statistics: Voxelwise analysis of multi-subject diffusion data. Neuroimage.

[19] Inglese M., Makani S., Johnson G., Cohen B.A., Silver J.A., Gonen O., Grossman R.I. (2005). Diffuse axonal injury in mild traumatic brain injury: A diffusion tensor imaging study. J. Neurosurg..

[20] Association Psychiatric Association (2013). Diagnostic and Statistical Manual of Mental Disorders.

[21] Kohen-Raz R. (1991). Application of tetra-ataxiametric posturography in clinical and developmental diagnosis. Percept. Mot. Skills.

[22] Smith S.M., Jenkinson M., Woolrich M.W., Beckmann C.F., Behrens T.E., Johansen-Berg H., Bannister P.R., De Luca M., Drobnjak I., Flitney D.E. (2004). Advances in functional and structural MR image analysis and implementation as FSL. Neuroimage.

[23] Behrens T.E., Woolrich M.W., Jenkinson M., Johansen-Berg H., Nunes R.G., Clare S., Matthews P.M., Brady J.M., Smith S.M. (2003). Characterization and propagation of uncertainty in diffusion-weighted MR imaging. Magn. Reson. Med..

[24] Smith S.M., Nichols T.E. (2009). Threshold-free cluster enhancement: Addressing problems of smoothing, threshold dependence and localisation in cluster inference. Neuroimage.

[25] Song S.K., Yoshino J., Le T.Q., Lin S.J., Sun S.W., Cross A.H., Armstrong R.C. (2005). Demyelination increases radial diffusivity in corpus callosum of mouse brain. Neuroimage.

[26] Mac Donald C.L., Dikranian K., Song S.K., Bayly P.V., Holtzman D.M., Brody D.L. (2007). Detection of traumatic axonal injury with diffusion tensor imaging in a mouse model of traumatic brain injury. Exp. Neurol..

[27] Kumar R., Macey P.M., Woo M.A., Harper R.M. (2010). Rostral brain axonal injury in congenital central hypoventilation syndrome. J. Neurosci. Res..

[28] Kumar R., Nguyen H.D., Macey P.M., Woo M.A., Harper R.M. (2012). Regional brain axial and radial diffusivity changes during development. J. Neurosci. Res..

[29] Song S.K., Sun S.W., Ramsbottom M.J., Chang C., Russell J., Cross A.H. (2002). Dysmyelination revealed through MRI as increased radial (but unchanged axial) diffusion of water. Neuroimage.

[30] Schmahmann J.D., Smith E.E., Eichler F.S., Filley C.M. (2008). Cerebral white matter: Neuroanatomy, clinical neurology, and neurobehavioral correlates. Ann. N.Y. Acad. Sci..

[31] Karlsgodt K.H., van Erp T.G.M., Poldrack R.A., Bearden C.E., Nuechterlein K.H., Cannon T.D. (2008). Diffusion tensor imaging of the superior longitudinal fasciculus and working memory in recent-onset schizophrenia. Biol. Psychiatry.

[32] Jang S.H. (2014). The corticospinal tract from the viewpoint of brain rehabilitation. J. Rehabil. Med..

[33] Jacobowitz D.M., Cole J.T., McDaniel D.P., Pollard H.B., Watson W.D. (2012). Microglia activation along the corticospinal tract following traumatic brain injury in the rat: A neuroanatomical study. Brain Res..

[34] Glickstein M. (1992). The cerebellum and motor learning. Curr. Opin. Neurobiol..

[35] Marsden J., Harris C. (2011). Cerebellar ataxia: Pathophysiology and rehabilitation. Clin. Rehabil..

[36] Jang S.H., Yi J.H., Kwon H.G. (2016). Injury of the inferior cerebellar peduncle in patients with mild traumatic brain injury: A diffusion tensor tractography study. Brain Inj..

[37] Drijkoningen D., Caeyenberghs K., Leunissen I., Vander Linden C., Leemans A., Sunaert S., Duysens J., Swinnen S.P. (2015). Training-induced improvements in postural control are accompanied by alterations in cerebellar white matter in brain injured patients. NeuroImage Clin..

[38] Hong J.H., Kim O.L., Kim S.H., Lee M.Y., Jang S.H. (2009). Cerebellar peduncle injury in patients with ataxia following diffuse axonal injury. Brain Res. Bull..

[39] Kwon H.G., Jang S.H. (2012). The usefulness of diffusion tensor imaging in detection of diffuse axonal injury in a patient with head trauma. Neural Regen. Res..

[40] Delouche A., Attye A., Heck O., Grand S., Kastler A., Lamalle L., Renard F., Krainik A. (2016). Diffusion MRI: Pitfalls, literature review and future directions of research in mild traumatic brain injury. Eur. J. Radiol..

[41] Qin W., Shui Yu C., Zhang F., Du X.Y., Jiang H., Xia Yan Y., Cheng Li K. (2009). Effects of echo time on diffusion quantification of brain white matter at 1.5 T and 3.0 T. Magn. Reson. Med..

[42] Smith D.H., Nonaka M., Miller R., Leoni M., Chen X.H., Alsop D., Meaney D.F. (2000). Immediate coma following inertial brain injury dependent on axonal damage in the brainstem. J. Neurosurg..

