# Supplementary material for: Altered White Matter Integrity after Mild to Moderate Traumatic Brain Injury
Source: J Clin Med. 2019 Aug 27;8(9):1318. doi: 10.3390/jcm8091318 (PMC6780936; doi:10.3390/jcm8091318)
Supplement: Supplementary file 1 [file jcm-08-01318-s001.pdf]

Supplementary Materials

Table S1. Results of TBSS analyses comparing RD values for the whole brain of the two groups.

Contrast	Voxel coordinates of Local maxima (MNI coordinates)			Side	Voxels	White Matter Tract	
	**x**	**y**	**z**			**JHU-WM Tractography Atlas**	**JHU-ICBM-DTI-81 WM Labels**
CON > PAT							
	27	-61	-36	R	43	Middle cerebellar peduncle	Corticospinal tract
	-17	-63	-31	L	28	Unclassified	
	-40	-51	10	L	16	Superior longitudinal fasciculus	Superior longitudinal fasciculus
	28	-55	-28	R	16	Unclassified	
	32	-54	-12	R	14	Unclassified	Inferior fronto-occipital fasciculus
	-25	-71	25	L	14	Unclassified	Inferior longitudinal fasciculus/ Forceps major
	8	-28	24	R	14	Body of corpus callosum	Anterior thalamic radiation
	25	-20	30	R	14	Superior corona radiata	Corticospinal tract
	13	-8	-7	R	12	Cerebral peduncle R	Anterior thalamic radiation
	-8	-57	-30	L	12	Unclassified	
	-12	-57	-26	L	11	Unclassified	Corticospinal tract/ Anterior thalamic radiation
	-15	-43	23	L	11	Splenium of corpus callosum	
PAT > CON							
	5	-22	-30	R	20	Corticospinal tract	Corticospinal tract
	-27	18	22	L	19	Anterior corona radiata	Uncinate fasciculus
	-39	7	-28	L	16	Unclassified	Uncinate fasciculus
	-19	47	4	L	15	Unclassified	Forceps minor
	-32	-16	-10	L	13	Fornix (cres) / Stria terminalis (can not be resolved with current resolution)	Anterior thalamic radiation
	-45	-55	-7	L	13	Unclassified	

30	44	-4	R	13	Unclassified	Uncinate fasciculus
-21	-43	45	L	13	Unclassified	Superior longitudinal fasciculus

MNI indicates Montreal Neurological Institute PAT indicates patients and CON indicates controls. Statistical significance was set at $p < 0.005$ with a cluster of an extent threshold of $k > 10$ voxels.

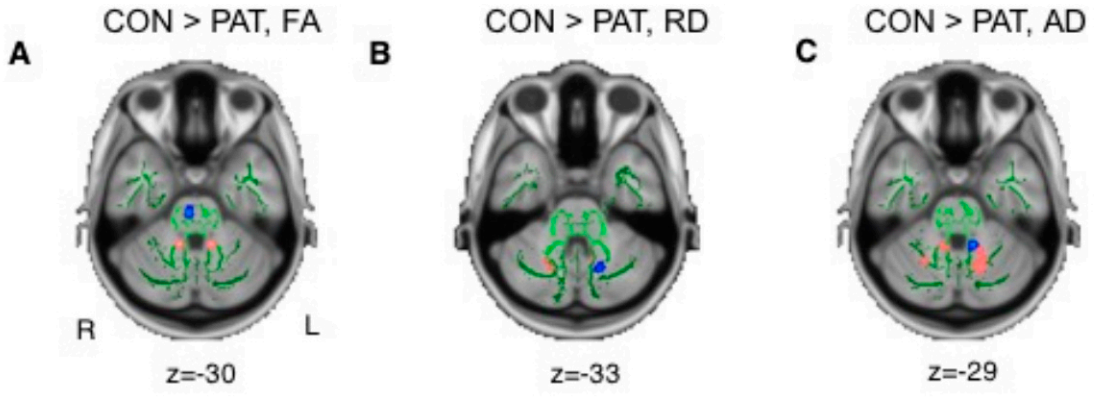

Figure S1. The significantly different diffusion-tensor anisotropy findings observed in the traumatic brain injury patients compared to the controls. The figures were visualized on a standard MNI152_T1 brain template with white matter skeleton (shown in green). The location visualized as blue (uncorrected $p < 0.001$ with a cluster of an extent threshold of k > 10 voxels) and pink (uncorrected $p < 0.005$ with a cluster of an extent threshold of k > 10 voxels) indicates the regions, such as the (**A**) right corticospinal tract and bilateral inferior cerebellar peduncles, (**B**) bilateral middle cerebellar peduncles, and (**C**) bilateral inferior cerebellar peduncles. Abbreviations: CON, controls; PAT, patients; FA, fractional anisotropy; RD, radial diffusivity; AD, axial diffusivity; R, right hemisphere; L, left hemisphere.

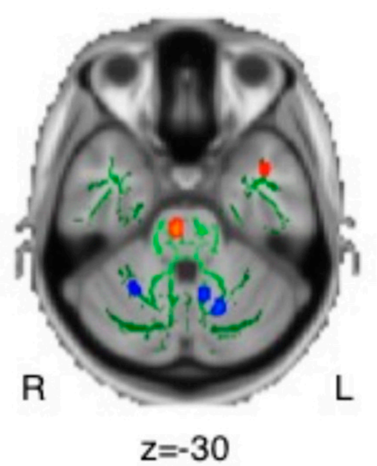

Figure S2. Significantly increased and decreased radial diffusivity observed in traumatic brain injury patients compared to controls (uncorrected $p < 0.005$ with a cluster of an extent threshold of $k > 10$ voxels). The data were visualized on a standard MNI152_T1 brain template with white matter skeleton (green). The location was set (z=-30) to visualize the increased radial diffusivity of the right corticospinal tract. The red represents the area where increased radial diffusivity was observed in the patient group while the blue represents the areas where decreased radial diffusivity was observed in the patient group; R, right hemisphere; L, left hemisphere.
